# Comparative Genomics of *Thiohalobacter thiocyanaticus* HRh1^T^ and *Guyparkeria* sp. SCN-R1, Halophilic Chemolithoautotrophic Sulfur-Oxidizing *Gammaproteobacteria* Capable of Using Thiocyanate as Energy Source

**DOI:** 10.3389/fmicb.2019.00898

**Published:** 2019-05-01

**Authors:** Stanislav I. Tsallagov, Dimitry Y. Sorokin, Tamara V. Tikhonova, Vladimir O. Popov, Gerard Muyzer

**Affiliations:** ^1^Bach Institute of Biochemistry, Research Centre of Biotechnology, Russian Academy of Sciences, Moscow, Russia; ^2^Winogradsky Institute of Microbiology, Research Centre of Biotechnology, Russian Academy of Sciences, Moscow, Russia; ^3^Department of Biotechnology, Delft University of Technology, Delft, Netherlands; ^4^Microbial Systems Ecology, Department of Freshwater and Marine Ecology, Institute for Biodiversity and Ecosystem Dynamics, University of Amsterdam, Amsterdam, Netherlands

**Keywords:** cyanate, halophiles, hypersaline lakes, thiocyanate dehydrogenase, sulfide

## Abstract

The genomes of *Thiohalobacter thiocyanaticus* and *Guyparkeria* (formerly known as *Halothiobacillus*) sp. SCN-R1, two gammaproteobacterial halophilic sulfur-oxidizing bacteria (SOB) capable of thiocyanate oxidation via the “cyanate pathway”, have been analyzed with a particular focus on their thiocyanate-oxidizing potential and sulfur oxidation pathways. Both genomes encode homologs of the enzyme thiocyanate dehydrogenase (TcDH) that oxidizes thiocyanate via the “cyanate pathway” in members of the haloalkaliphilic SOB of the genus *Thioalkalivibrio.* However, despite the presence of conservative motives indicative of TcDH, the putative TcDH of the halophilic SOB have a low overall amino acid similarity to the *Thioalkalivibrio* enzyme, and also the surrounding genes in the TcDH locus were different. In particular, an alternative copper transport system *Cus* is present instead of *Cop* and a putative zero-valent sulfur acceptor protein gene appears just before TcDH. Moreover, in contrast to the thiocyanate-oxidizing *Thioalkalivibrio* species, both genomes of the halophilic SOB contained a gene encoding the enzyme cyanate hydratase. The sulfur-oxidizing pathway in the genome of *Thiohalobacter* includes a Fcc type of sulfide dehydrogenase, a rDsr complex/AprAB/Sat for oxidation of zero-valent sulfur to sulfate, and an incomplete Sox pathway, lacking SoxCD. The sulfur oxidation pathway reconstructed from the genome of *Guyparkeria* sp. SCN-R1 was more similar to that of members of the *Thiomicrospira-Hydrogenovibrio* group, including a Fcc type of sulfide dehydrogenase and a complete Sox complex. One of the outstanding properties of *Thiohalobacter* is the presence of a Na^+^-dependent ATP synthase, which is rarely found in aerobic Prokaryotes.Overall, the results showed that, despite an obvious difference in the general sulfur-oxidation pathways, halophilic and haloalkaliphilic SOB belonging to different genera within the *Gammaproteobacteria* developed a similar unique thiocyanate-degrading mechanism based on the direct oxidative attack on the sulfane atom of thiocyanate.

## Introduction

Chemolithoautotrophic sulfur-oxidizing bacteria (SOB) play an important role in the global biogeochemical sulfur cycle by oxidizing various reduced sulfur compounds, such as sulfide, sulfur, thiosulfate and polythionates, to sulfate both in natural and industrial habitats (Janssen et al., 2009; Caia et al., 2017; Wasmund et al., 2017).

Only a few of these SOB have the ability to use thiocyanate as an energy source. Thiocyanate can be regarded as an inorganic nitrile whereby its carbon atom is linked to a reduced sulfur atom (N≡C-S^-^). This reduced sulfur compound is forming at low concentrations in natural habitats by reaction of cyanide with inorganic sulfan atom donors, such as thiosulfate, tetrathionate or polysulfide (Kurashova et al., 2017). In addition, thiocyanate is formed as a major waste product in metallurgy and precious metal mining with cyanide (Luque-Almagro et al., 2016). Since it has a reduced sulfane atom similar to sulfide, it can be used by some SOB as an electron donor. However, primary degradation of the thiocyanate molecule is needed before the reduced sulfane atom could be utilized, which is not an easy task, since the molecule is chemically stable, which might be a reason for the scarcity of thiocyanate-utilizing SOB known in culture.

Currently, two hydrolytic pathways for the primary degradation of thiocyanate are suggested in SOB, i.e., either (i) hydrolysis of the nitrile (N≡C) bond, or (ii) hydrolysis of the C-S bond (Kelly and Baker, 1990). The first reaction results in formation of carbonyl sulfide (O = C = S) and ammonia for which the enzyme *thiocyanate hydrolase*, a cobalt-containing analog of organic nitrile hydratases, is responsible (Katayama et al., 1998). This enzyme is found in freshwater beta- and haloalkaliphilic gamma-proteobacterial SOB, such as *Thiobacillus thioparus* (Katayama et al., 1992), *Thioalkalivibrio thiocyanodenitrificans* (Berben et al., 2017b) and *Thiohalophilus thiocyanoxidans* (Bezsudnova et al., 2007). Recently, it has also been identified in metagenomes of a thiocyanate-degrading bioreactor community (Rahman et al., 2017). The second pathway leads to formation of cyanate (N = C-O^-^) and sulfide and its existence was predicted on the basis of a presence of activity of enzyme cyanate hydratase (cyanase) in thiocyanate-utilizing *Thiobacillus* species (Youatt, 1954). However, it is now clear that cyanase is not a reliable marker for this pathway. Work on haloalkaliphilic thiocyanate-oxidizing SOB from the genus *Thioalkalivibrio* had demonstrated that cyanase activity was absent in these SOB, despite a definite operation of this “cyanate pathway” of thiocyanate degradation, proven by the cyanate formation as an intermediate (Sorokin et al., 2001). Furthermore, cyanase genes are present in a large number of genomes of heterotrophic bacteria not related to the known SOB and, most probably, the encoded proteis have a function of either cyanate detoxification (as in the case of cyanide-degrading heterotrophs) (Park et al., 2017), or as a source of ammonia, either for nitrogen assimilation, or as energy source for ammonia-oxidizing archaea (Palatinszky et al., 2015).

Another discrepancy from the previously proposed hydrolytic mode of the “cyanate pathway” is that this reaction is not hydrolytic in *Thioalkalivibrio*. In whole cells it is possible only in presence of oxygen, while in cell homogenates high potential electron acceptors, such as cytochrome *c*550 is needed. The products of the reaction are cyanate and elemental sulfur, instead of sulfide as was believed previously (Sorokin et al., 2001). This indicates that the mechanism of the reaction is not hydrolysis, as in the case of thiocyanate hydrolase, but rather a direct oxidation of the sulfane atom. Cells of *Thioalkalivibrio* strains grown with thiocyanate overproduced (in comparison to cells grown with thiosulfate) a soluble periplasmic single-subunit protein (56 kDa) apparently responsible for this reaction. A recent study has demonstrated that the gene encoding this protein was among the highest expressed in *Thioalkalivibrio thiocyanoxidans* ARh2^T^ cells grown in thiocyanate-limited chemostat culture in contrast to cells grown with thiosulfate (Berben et al., 2017a). The purified protein, named thiocyanate dehydrogenase (TcDH), contains copper in its active center and catalyzes oxidative degradation of thiocyanate into cyanate and sulfur in the presence of oxidized cytochrome *c*550 or ferricyanide (unpublished results).

**Figure 1:**
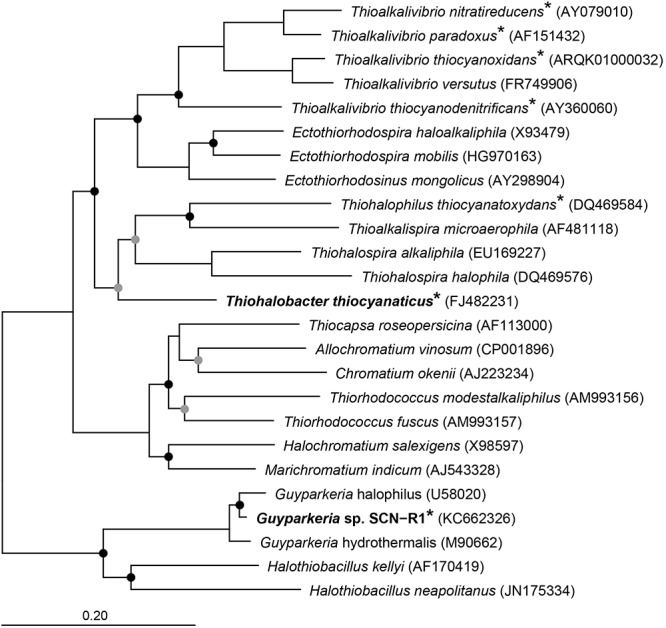
Neighbor joining tree based on 16S rRNA sequences showing the phylogenetic affiliation of *Thiohalobacter thiocyanaticus* and *Guyparkeria* sp. SCN-R1 in comparison to other sulfur and/or thiocyanate-oxidizing species of the *Gammaproteobacteria.* Species that have been demonstrated to oxidize thiocyanate are indicated by an asterisk. The circles on the nodes indicate bootstrap values between 100–75% (black dots) and between 75–50% (gray dots) out of 1000 iterations. The scale bar indicates sequence differences. Sequences of different members of the *Thermotogales* were used as an outgroup, but were pruned from tree.

Apart from the haloalkaliphilic species of the genus *Thioalkalivibrio* oxidizing thiocyanate via the “cyanate pathway,” we have also isolated and characterized two halophilic gammaproteobacterial SOB utilizing thiocyanate as the electron donor with the intermediate formation of cyanate. *Thiohalobacter thiocyanaticus* HRh1^T^ was enriched and isolated from hypersaline lakes in south-western Siberia with thiocyanate as substrate and NaCl concentration of 1–2 M (Sorokin et al., 2010), while *Guyparkeria* (former *Halothiobacillus*) sp. strain SCN-R1 was recovered from a thiocyanate-degrading bioreactor operating at moderate salinity of 0.5–1 M NaCl (Sorokin et al., 2014). They are associated with two different clusters of SOB within the *Gammaproteobacteria* (Figure 1). In this paper we present the results of a comparative analysis of the genomes of these two thiocyanate-oxidizing halophilic SOB with a particular focus on their sulfur metabolism.

## Materials and Methods

### Cultivation Conditions

*Thiohalobacter thiocyanaticus* and *Guyparkeria* sp. strain SCN-R1 were both grown in 200 ml mineral medium with 20 mM thiosulfate as substrate. The medium contained 1 M NaCl, 3 g l^-1^ potassium phosphate buffer, pH 7, 1 mM MgCl_2_, 1 ml l^-1^ of trace metal solution (Pfennig and Lippert, 1966), 10 μg l^-1^ of vitamin B_12_ and 4 mM NH_4_Cl. To prevent acidification of the medium during thiosulfate oxidation, 40 mM of filter-sterilized NaHCO_3_ solution was added. The cultures were incubated in 1 L flasks closed with a rubber septum on a rotary shaker at 140 rpm and 30°C. After complete consumption of thiosulfate (monitored by acidic 0.01 N J_2_ titration), the cells were harvested by centrifugation, washed once in 1 M NaCl and stored at -80°C for DNA extraction.

Comparative denaturing gel electrophoresis with fractions of cell extract from cells of two halophilic SOB species grown with either thiosulfate or thiocyanate as electron donor were performed according to Laemmli protocol using a gradient of PAGE from 5 to 15% (Laemmli, 1970).

### Genome Sequencing, Assembly and Annotation

High molecular weight DNA was extracted by using the DNeasy UltraClean Microbial Kit (Qiagen). The extracted DNA was sequenced by the commercial company BaseClear (Leiden, Netherlands). The genome of *Guyparkeria* sp. strain SCN-R1 was sequenced with Illumina HiSeq, while the genome of *Thiohalobacter thiocyanaticus* was sequenced with a combination of Illumina HiSeq and PacBio Sequel. *De novo* assembly of the Illumina HiSeq paired-end sequence reads was performed with SPAdes. SSPACE Premium Scaffolder version 2.3 (Boetzer et al., 2011) was used to place the assembled contigs into scaffolds. Gapped regions within the scaffolds were (partially) closed using GapFiller version 1.10 (Boetzer and Pirovano, 2012). *De novo* assembly of the PacBio reads was performed using HINGE (Kamath et al., 2017). Mis-assemblies and nucleotide disagreements between the assembled sequences and the Illumina data were corrected with Pilon version 1.21 (Walker et al., 2014). The completeness of the genomes was determined with CheckM (Parks et al., 2014). The assembled genomes were first annotated in RAST (Overbeek et al., 2014^1^) and subsequently in Genbank. An overview of the locus tags, annotation, cellular location, presence of transmembrane helices, etcetera of translated a sequences of *Guyparkeria* sp. SCN-R1 have been given in Supplementary Table S1, and that of *Thiohalobacter thiocyanaticus* Hrh1^T^ in Supplementary Table S2. The genome sequences have been deposited at Genbank under accession number QZMT00000000 for *Guyparkeria* sp. SCN-R1 and QZMU00000000 for *Thiohalobacter thiocyanaticus* HRh1^T^.

### Comparative Sequence Analysis

Search of TcDH and other genes in bacterial genomes was performed using Tblastn algorithm in standalone BLAST v. 2.7.1 (Camacho et al., 2009) using a single genome sequence as database. Search of homologs was performed by online BLAST using BLASTp and PHI-BLAST algorithms (2017) with default settings (NCBI Resource Coordinators, 2017). InterProScan online tool and CDD/SPARCLE database was used for hypothetical protein function (Marchler-Bauer et al., 2017). Secondary structure prediction was performed with JPred4 (Drozdetskiy et al., 2015) with default settings. Jobs were submitted via JalView v. 2.10.3 (Waterhouse et al., 2009).

Transmembrane helix predictions: TMHMM2^2^ – one of the recommended predicting tools according to Punta et al. (2007). Signal peptide prediction was performed with signalP v. 4.1 (Petersen et al., 2011).

Multiple alignments of protein sequences: Blastp search results with low *e*-values (<10^-5^) and reasonable sequence identity (>30%) was used as inputs for ProbCons algorithm (Do et al., 2005).

For global pairwise alignment Needleman-Wunch algorithm on NCBI website^3^ was used with default parameters.

Comparative analysis of the ATPase sequences was done with the online program www.phylogeny.fr using the Advanced Mode. The sequence logo’s was created with the web-based application WebLogo (Crooks et al., 2004).

For a whole genome comparison of *Thiohalobacter thiocyanaticus* HRh1^T^ with a closely related *Thiohalobacter* strain FOKN1, the two genomes were aligned by using Mauve 2.3.1 (Darling et al., 2004) plugin inside Geneious 7.1 software (Biomatters Ltd., New Zealand). The two scaffolds of HRh1^T^ and the chromosome of FOKN1 genomes were previously rearranged in order to obtain the correct alignment (Consensus 1 of HRh1^T^ was reverse-complemented before concatenate with HRh1^T^ Consensus 0; FOKN1 was reverse-complemented; both residue numbering changed to start with the chromosome replication initiator DnaA gene). Average nucleotide identity (ANI) was calculated using the BLAST (ANIb) and MUMmer (ANIm) algorithms performed by the JSpecies online tool (Richter and Rosselló-Móra, 2009^4^), while digital DNA-DNA hybridization (dDDH) was performed by Genome-to-Genome Distance Calculator 2.1 online submission form (GGDC, Meier-Kolthoff et al., 2013^5^).

### Phylogenetic Analysis

Phylogenetic analysis of 16S rRNA sequences was done in ARB (Westram et al., 2011) using the SILVA database (Quast et al., 2013). Briefly, the 16S rRNA sequences of *Thiohalobacter thiocyanaticus* HRh1^T^ and *Guyparkeria* sp. SCN-R1 were aligned using SINA (Pruesse et al., 2012). The aligned sequences were imported into ARB and added to an existing tree using the parsimony approach. Subsequently, a neighbor joining tree was calculated using 16S rRNA sequences of related SOB.

SoeA-like sequences were retrieved from the NCBI protein database, by using protein BLAST. Sequences were aligned using T-COFFEE (Di Tommaso et al., 2011) and the most likely amino acid substitution matrix was determined using protest 3 LG model (Abascal et al., 2005; Le and Gascuel, 2008) with gamma-distributed rates and empirical frequencies (model parameter –m PROTGAMMALGF in RAxML) for all alignments (Stamatakis, 2014, 2015). Maximum likelihood trees were calculated using RAxML 8.2.12 using the rapid bootstrap analysis algorithm. The tree was visualized in MEGA7.

## Results and Discussion

### General Genome Features

Table 1 gives an overview of the general genome features of *Guyparkeria* sp. SCN-R1 and *Thiohalobacter thiocyanaticus* HRh1^T^. The genome of *Guyparkeria* sp. SCN-R1 consists of 68 scaffolds with a total size of 2.4 Mbp. CheckM analysis showed that it was 100% complete. The genome of *Thiohalobacter thiocyanaticus* HRh1^T^, which was sequenced with PaqBio and Illumina, consists of two scaffolds with a total size of 3.3 Mbp. CheckM analysis showed a completeness of 97%. Supplementary Tables S1, S2 show a selection of the most important functional proteins found in two genomes.

**Table 1:** Genome statistics.

	*Guyparkeria* sp.	*Thiohalobacter*
Parameters	SCN-R1	*thiocyanaticus* HRh1^T^
Genome size (bp)	2,418,072	3,266,530
Number of scaffolds	68	2
N50	128,555	3,056,480
% Completeness	100	97
% Contamination	0.57	1.41
% GC	64.7	64.3
Number of coding sequences	2183	2843
Number of RNAs	50	45

### Sulfur Oxidation Pathways

The genomes of both organisms contain genes encoding sulfide dehydrogenase of the Fcc (flavocytochrome *c*) type, although in both cases the cytochrome *c* subunit has only one heme *c* motif instead of the customary two motifs. Such modification had also been noticed for haloalkaliphilic SOB species of the genus *Thioalkalivibrio* (Berben et al., 2017a,b). The catalytic flavin subunit of *Thiohalobacter* is only distantly related to that of *Thioalkalivibrio*, while that of *Guyparkeria* is close to the one present in *Halothiobacillus neapolitanus*. Genes for the alternative sulfide dehydrogenase – sulfide quinone reductase (SQR) were not found in both genomes.

Both organisms have the Sox system, but in a different assemblage. The genome of *Guyparkeria* encodes a complete Sox pathway consisting of *soxABXYZCD*, allowing the oxidation of both sulfur atoms of thiosulfate completely to sulfate, while *Thiohalobacter* has an incomplete Sox system, i.e., lacking the *soxCD* sulfur dehydrogenase, which is more typical for the gammaproteobacterial SOB. Although at optimal growth conditions with thiosulfate as substrate HRh1^T^ did not produce any visible zero-valent sulfur particles, extracellular sulfur started to accumulate in cultures when pH dropped below 6 and at oxygen limitation. Also, young colonies on thiosulfate agar were always silver-white due to extracellular sulfur formation and the sulfur disappeared only in a month-old colonies (Sorokin et al., 2010). The zerovalent sulfur is further oxidized to sulfite by the reverse Dsr system. In *Thiohalobacter* the rDsr system is represented by two gene clusters located in the vicinity of each other: the functional *dsrAB* (related to both aerobic and anaerobic SOB within the order *Chromatiales*) and the accessory *dsrMKJOP*. The *dsrEFH*, which is needed for the transfer of zerovalent sulfur atom into the cell and normally present in the rDsr system, is absent. Three *dsrC/tusE* sulfur transferase genes were present elsewhere in the genome of *Thiohalobacter*. While one of them is definitely a genuine *dsrC*, important for the rDsr function, the two other copies might fulfill the function of the missing DsrEFH. The indicator of the direct pathway, *dsrD* (Rabus et al., 2015), is absent in the genome of *Thiohalobacter*. Therefore, it still can be considered as a variation of the rDSR system.

Further oxidation of sulfite formed by the action of rDSR system in *Thiohalobacter* can be executed by two pathways. One is traditionally associated with the rDsr and includes adenylyl phosphosulfite reductase (Apr) and sulfate adenylyl transferase (Sat). The gene cluster including putative AprABM and a separate *sat* gene are present in the *Thiohalobacter* genome. A second pathway includes direct sulfite oxidation to sulfate by a putative SoeABC quinone-interacting sulfite dehydrogenase which is encoded by an operon whereby the catalytic subunit is annotated as DMSO reductase or formate dehydrogenase. SoeA, DmsA, and FdhA all belong to the Mo oxidoreductase superfamily and, therefore, phylogenetic reconstruction is necessary to predict the most probably function. This done, the translated protein sequence from *Thiohalobacter* indeed clustered with a large group of putative SoeA present in the genomes of lithoautotrophic SOB (Supplementary Figure S1). However, the role of this gene cluster in sulfite oxidation can only be confirmed by expression analysis. Currently, the only functional evidence on the importance of this alternative type of sulfite dehydrogenase (to its more studied SorAB homologous enzyme which catalytic subunit also belongs to the Mo-supurfamily oxido-reductases and which uses cytochrome *c* as its immediate *e*-acceptor) has been provided for anoxygenic SOB *Allochromatium vinosum* (Dahl et al., 2013), whereby it also coexists with the rDSR system.

**Figure 2:**
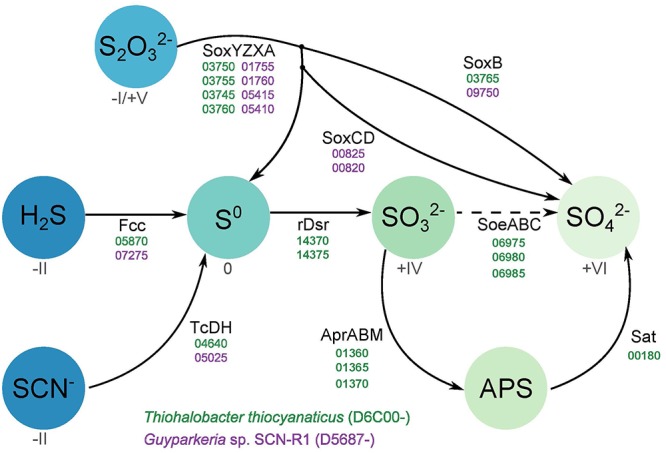
Overview of the different sulfur oxidation genes present in *Thiohalobacter thiocyanaticus* and *Guyparkeria* sp. SCN-R1. Dash arrow – hypothetical reaction.

Figure 2 gives an overview of the different sulfur oxidation-related genes present in both *Thiohalobacter thiocyanaticus* HRh1^T^ and *Guyparkeria* sp. SCN-R1.

### Inorganic Carbon Fixation

Both organisms encode form IAc RuBisCO (“green form”), indicating the presence of the Calvin-Benson ribulose-bisphosphate carboxylase pathway for autotrophic CO_2_ fixation. The large subunit CbbL of *Thiohalobacter* is closely related to the *Thioalkalivibrio* CbbL proteins, while the CbbL of *Guyparkeria* is equally related to both *Thioalkalivibrio* and *Halothiobacillus neapolitanus*. The RuBisCO operon of *Guyparkeria*, in addition, has genes encoding carboxysome shell proteins (Cso), which is a common feature for this phylogenetic radiation of SOB (Yeates et al., 2008).

Another important group of enzymes in CO_2_ fixation in autotrophic prokaryotes are carbonic anhydrases (CA), interconverting CO_2_ and bicarbonate. Both genomes contain (independently placed from the RuBisCO operon) a γ-CA. In addition, *Thiohalobacter* also have two different copies of β-CA. Larger assortment of the CA in *Thiohalobacter* is probably related to the absence of carboxysomes that play an additional role in the carbon fixation efficiency, i.e., the carboxysomal shell protein CsoS3 is functioning as the carboxysome-specific carbon anhydrase (Price et al., 2008). Moreover, each organism has another CA copy, which is associated with the bicarbonate-dependent cyanate hydratase, i.e., an α-CA in *Thiohalobacter* and a β-CA in *Guyparkeria*.

### Bioenergetics

Both halophilic SOB encode two types of heme-copper terminal oxidases, i.e., a cytochrome *c* oxidase of the *cbb_3_* type, which is regarded as a high O_2_ affinity enzyme, and a quinol oxidase of the *ba_3_* type. In addition, the *Thiohalobacter* genome also contains an operon encoding for a second quinol oxidase of the *bo_3_* type, belonging to the copper-free cytochrome *b* class of oxidases. Membrane spectroscopy showed a clear difference in the heme composition between thiosulfate and thiocyanate-grown cells of *Thiohalobacter*: in the former hemes *c*, *b*, and *a_3_* types were visible with a domination of the heme *c*, while in the thiocyanate-grown cells only hemes *c* and *b* were detected with a much higher proportion of heme *b* (Supplementary Figure S2). This might indicate that during growth with thiosulfate at least two oxidases, *cbb_3_* and *ca_3_*, were expressed, while during growth on thiocyanate only the *cbb_3_* was expressed.

**Figure 3:**
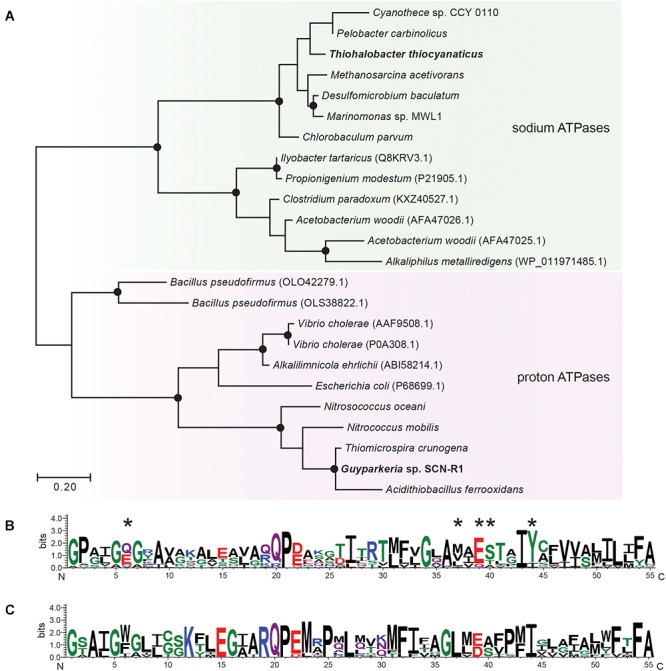
Maximum likelihood tree **(A)** and sequence logos of sodium **(B)** and proton-dependent **(C)** ATP synthases (C-subunit). The sequences of *Gyparkeria* sp. SCN-R1 and *Thiohalobacter thiocyanaticus* HRh1^T^ are written in bold type. The sodium ligands are indicated with asterisks including the Na^+^-binding motif ESTxxY (see Mulkidjanian et al., 2008). The black dots on the tree indicate bootstrap values between 100 and 75% out of 100 iterations.

For ATP synthesis, *Guyparkeria* genome encodes an H^+^-dependent F_1_F_0_ ATP synthase, which is normally present in aerobic chemolithotrophic SOB. In contrast, the genome of *Thiohalobacter* contains two operons encoding different types of ATPases. One is a 4-subunit H^+^-dependent V type ATP-synthase, and another one is an apparent sodium pumping F_1_F_0_ ATP synthase (Figure 3 and Supplementary Figure S3) (with the typical operon structure *atpDCIRBEFAG* and the unique Na^+^-binding motif EST-XX-Y, that is normally present as a secondary ATPase to its H^+^-dependent analog functioning for Na^+^ extrusion (Dibrova et al., 2010). Interestingly, however, in *Thiohalobacter* the Na^+^-ATP synthase partners with a H^+^-V-type ATPase, which is rare in *Proteobacteria*.

For the Na^+^/H^+^ homeostasis, both SOB encode the multi-sub-unit Na^+^/H^+^ antiporter *MnhABCDEFH*, although the operon of *Thiohalobacter* seems lacking the subunit C.

### Osmoregulation

Both organisms belong to moderate halophiles that usually cope with osmotic stress by synthesizing (or importing) organic osmolytes. The genome of *Thiohalobacter* encodes genes for *de novo* synthesis of three potential osmolytes, i.e., trehalose, sucrose and glycine betaine. In contrast, *Guyparkeria* seems to rely on the synthesis of ectoin as the most probable main osmolyte, and sucrose as the secondary compound, which it also can import. Both halophiles can also import glycine betaine, but for this *Guyparkeria* has a dedicated transporter *OpuD*, while *Thiohalobacter* encodes a less specialized ABC-type transporter for proline/glycine betaine.

### Thiocyanate Metabolism

Because both species are able to oxidize thiocyanate, either thiocyanate hydrolyase or TcDH have to be present. Comparative gel electrophoresis analysis of total protein extracts from cells grown with thiosulfate against thiocyanate revealed clear differences only in case of *Thiohalobacter*, whose profile on thiocyanate showed two heavily overproduced proteins, one of which had a similar mass to the TcDH protein of *Thioalkalivibrio* (∼54 kDa) (Supplementary Figure S4). Tblastn was used to search for TcDH and thiocyanate hydrolase sequences in the genomes of *Guyparkeria* SCN-R and *Thiohalobacter thiocyanaticus* HRh1^T^. Homologs of thiocyanate hydrolase were not found. However, a single copy of a TcDH homolog was found in both genomes. In addition, a short TcDH fragment corresponding to 52 amino acids was detected upstream of the TcDH gene in the genome of *Guyparkeria*. The fragment is not present in the Genbank annotation and can be detected only by protein sequence search against nucleotide sequence. It seems to be just a small duplicated non-functional DNA fragment from a TcDH gene.

**Figure 4:**
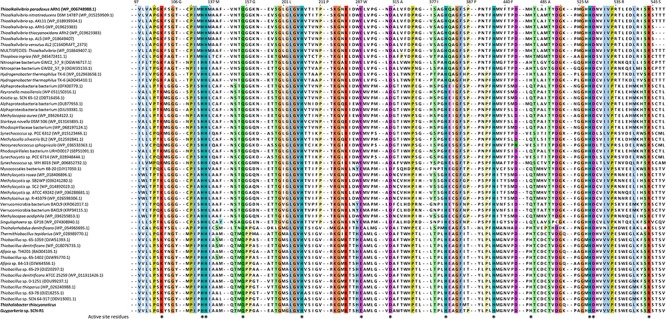
Fragment of a multiple alignment of TcDH sequences, containing residues of the enzyme’s active site marked by asterisks: Lys103, His135, His136, Gln156, His206, Glu288, Asp314, His381, His437, His482, His528, Asp529, Arg544. Color intensity is proportional to residue conservation. The top sequence corresponds to the model TcDH from *Thioalkalivibro paradoxus* ARh1 with known crystal structure (PDB ID 5F75), the last two sequences are TcDH homologs from *Thiohalobacter thiocyanaticus* HRh1^T^ and *Guparkeria* SCN-R1. Gaps between alignment fragments are represented by dashed lines.

Comparative analysis of the two sequences from halophilic SOB was performed with the TcDH from *Thioalkalivibro paradoxus* ARh1^T^ (crystal structure PDB id 5F75) and its various homologs found in the bacterial genomes (sequence identity above 30%). All residues of the active site in TcDH were present in both TcDH homologs of *Guyparkeria* and *Thiohalobacter* (Figure 4). These residues either coordinate copper ions and water molecules in the central cavity of the 5F75 structure, or form the shape of this cavity. The presence of all these highly conserved and structurally important residues in both sequences provide strong evidence that these genes encode TcDH. In addition to the presence of TcDH sequences, we also found cyanase operons in both genomes. However, for some reasons, the cyanase activity was not measurable in cell homogenates of both bacteria (Sorokin et al., 2010, 2014).

**Figure 5:**
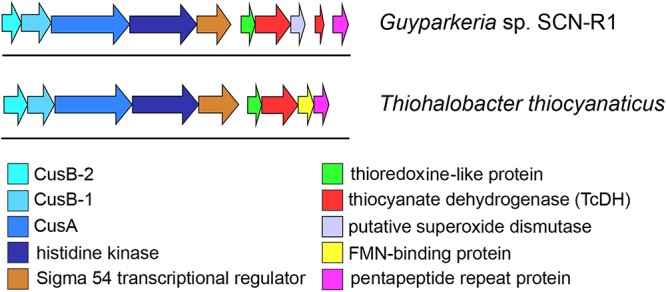
Genome region around TcDH gene in *Thiohalobacter thiocyanaticus* and *Guyparkeria* SCN-R1. Both organisms contain a number of similar genes around TcDH in the same order (colored arrows). “Blue” genes are likely to be related to a copper transport system (Cus).

The genome region around the TcDH genes was analyzed and manually annotated using various bioinformatic tools (Figure 5). To determine conserved genes for both bacteria each gene in this region was aligned against the other genome. Six genes upstream of the TcDH genes are common to both organisms: they have sequence identity of around 50%, and the same order (Figure 5).

### Sequence Analysis of Genes Around the TcDH Gene

A gene cluster upstream of the TcDH gene in *Guyparkeria* and *Thiohalobacter* encode proteins belonging to thioredoxin superfamily. However a conservative COX motif, which is critical for redoxin activity (Meyer et al., 2008), is absent in both sequences. Multiple alignment (Supplementary Figure S5) of these two sequences and their homologs have shown that a part of this superfamily members lack a COX motif or any other cysteines. As a consequence, they lack oxidoreductase activity, which, however, does not affect their ability to bind glutathione (Nardini et al., 2004). A predicted function of proteins incapable of glutathione oxidation is glutathione transport to a protein partner (Nardini et al., 2004). Multiple alignment also shows an appearance of COX motif in several sequences (black boxes), while the overall sequence in this region remains conserved (Supplementary Figure S3). A number of these COX-missing proteins was structurally characterized (Gaudet et al., 1996; Wang et al., 1998; Liepinsh et al., 2001) and have been shown to preserve a conserved tertiary structure of the thioredoxin-like domain. So, we can assume that the tertiary structure of all these sequences is similar and is suitable for the sulfur atom binding/carrying.

In another study, a complex of a thioredoxin-like protein with its protein partner was crystallized (Gaudet et al., 1996), which shows the ability of this family members to form a complex with other proteins. Interestingly, the partner of thioredoxin-like protein in that case (PDB id 2TRC) has exactly the same fold as TcDH – a 7 bladed beta-propeller. Since TcDH catalyzes sulfane atom oxidation of NCS^-^ to sulfur and NCO^-^, it needs an acceptor for the zero-valent sulfur atom. Thus, the protein encoded by the first gene upstream to TcDH might fulfill the role of accepting sulfur from TcDH and transporting it to enzymes involved in further oxidation of zero-valent sulfur atom to sulfate.

#### Sigma 54 Transcriptional Activator

The function of next upstream gene was revealed by BLASTp search using sequences from PDB as protein database. Several homologs were found with a moderate (40%) identity and good coverage. All of them were described as sigma 54 transcriptional regulators. Needleman-Wunch pairwise alignment with NtrC1 protein from the extreme thermophile *Aquifex aeolicus* (pdb id 1NY5) have shown reasonable identity (35%) and high score (545) (Fukayama et al., 1992). This result allows to assign similar function to this upstream TcDH gene. A presence of sigma 54 transcryptional regulator has also previously been shown in the TcDH operon of thiocyanate-oxidizing members of the genus *Thioalkalivibrio* (Berben et al., 2017a,b).

#### Copper Transport Genes of Cus System

Thiocyanate dehydrogenase has a Tat signal peptide and is exported to periplasm with twin-arginine translocation pathway. This pathway is used to transfer a folded protein to the periplasm. The reason for exporting an enzyme in 3D conformation is in acquiring a complex multi-atom cofactor in the cytoplasm (Lee et al., 2006), and in case of TcDH it is a multi-copper active site. That is why copper ions need to be transferred into cytoplasm. Also, bacteria need to supply TcDH synthesis with sufficient amount of copper. Therefore, it is not surprising to find genes encoding copper binding/transferring proteins close to TcDH. These genes belong to Cus system, which is known to be involved in copper homeostasis in *Escherichia coli*. as a copper-responsive regulatory system (Grass and Rensing, 2001).

Three genes are located further upstream to TcDH (Figure 5) with almost no gaps, indicating a possibility of their simultaneous expression and a common function. The first two of them encode membrane proteins. According to InterProScan classification they can be either an inner membrane ABC transporter HlyB and an outer-membrane channel protein TolC (Anes et al., 2015; Kim et al., 2016), or inner membrane CusA and outer membrane CusC proteins (Su et al., 2009). The latter are part of CusCBA copper transport system. CusA encodes copper membrane transporter efflux pump. This protein is reported to transport Cu(I) and Ag(I) ions across inner membrane in both directions (Long et al., 2010, 2012). Considering copper dependence of TcDH, these genes are more likely to be Cus genes homologs. Needleman-Wunch pairwise alignment of CusA sequence of *Guyparkeria* SCN-R1 and a CusA sequence of solved crystal structure (PDB ID 3K07) has 31% identity and high alignment score (1194) supporting the assumed copper transport function of the 4th gene of the TcDH operon. Based on that, we can assume that the other genes of the operon are also related to copper transport. One of them should be an outer member protein, another – a fusion protein, bridging this protein with CusA across periplasm.

The gene adjacent to *cusA* encodes a large 800 amino acid protein which was expected to be CusC, however, pairwise alignments with known CusC showed poor results. According to phi-blast search results and InterProScan classification this is a homolog of a histidine kinase. TMHMM2 analysis detects 8 transmembrane helixes with high confidence at 23–269 residues (Supplementary Figure S6). This transmembrane domain was also detected by InterProScan and confirmed by secondary structure prediction with JPred. The transmembrane domain of this protein is not assigned to any protein family. Multiple alignment shows presence of highly conserved methionines and histidines in this domain, that are typical protein ligands for copper ions (Rubino and Franz, 2012). Residues 280–620 were determined as GAF-like domain, and 640–849 as a histidine kinase family. GAF domain has regulating function: binding cAMP or cGMP to that domain up-regulate activity (Heikaus et al., 2009). The remaining histidine kinase domain can perform various functions. In several studies Ag and Cu transport activity was reported for proteins with this domain (Gudipaty and McEvoy, 2014; Affandi et al., 2016). Overall, we can say that this is a membrane-bound protein with a putative copper transport function (same function as *cusC*).

**Figure 6:**
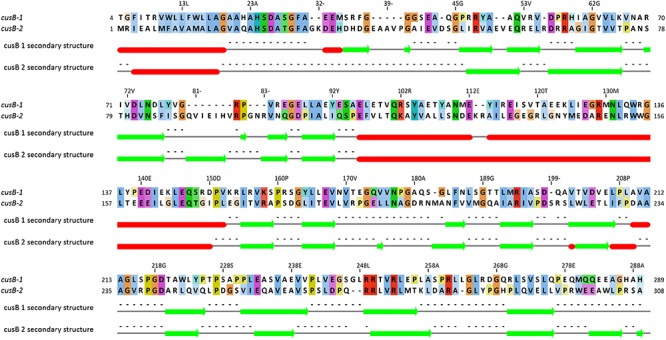
Pairwise alignment of CusB-1 and CusB-2 protein sequences from *Thiohalobacter thiocyanaticus* perfomed by Needleman-Wunch algorithm (sequences identity 28%, similarity 43%, and alignment score 186). Secondary structure of each protein was modeled by Jpred 4. Beta-strands and alpha helices are shown by green arrows and red cylinders, respectively.

The first two genes (5th and 6th upstream to TcDH) in *cus* operon (*cusB1* and *cusB2*) encode two proteins of HylD superfamily. According to superfamily description, these proteins form a bridge between inner and outer membrane transporters. These genes were also found in a few other bacterial genomes (*Thiobacillus thioparus* NZ_KB891333.1, *Hydrogenophilales* bacterium, *Thiobacillus denitrificans* NC_007404.1, *Thermithiobacillus tepidarius*). In case of these organisms, if one *cusB* gene is present in a genome, it is always followed by the second *cusB*. Moreover, this gene pair was always preceded by *cusA* gene. Each encoded protein of this pair has a high pairwise alignment score (600+) and moderate sequence similarity with its homologs from other species (Figure 6): for example CusB1 – 36% for WP_018509489.1, 37% for WP_011311413.1; CusB2 – 39% WP_011311412.1, 41% for WP_018509490.1. However, sequence identity and alignment score between two proteins from the same organism is much lower (24%). Despite of that, the proteins have a number of similarities, and the most striking one is that they apparently belong to the same protein family, determined by InterProScan. Both have a signal peptide at 20–25 AA. Prediction analysis with JPred 4 demonstrated a very similar secondary structure pattern. After a signal sequence the 6 β-strands (residues 35–92 in cusB1 and 52–110 in cusB2) is present, followed by a α-helix domain (114–169 in cusB1 and 96–153). The remaining part of both proteins has β-strands structure with one small helix containing 14 strands in cusB1 and 8 strands in cusB2. Most of these strands have a lot of conserved residues, while other parts of the sequence are more variable. This secondary structure closely coincides with β-strands and α-helix sequences and length of cusB crystal structure (Su et al., 2009). These similarities might be taken as evidence of a common function of both proteins, but the reason of such duplication is unclear.

In the previously analyzed genomes of haloalkaliphilic thiocyanate-oxidizing members of the genus *Thioalkalivibrio* the TcDH gene is flanked by *copC* and *copD* encoding copper-binding/transporting proteins. However, these genes were not found in halophilic *Thiohalobacter* and *Guyparkeria*, while obviously being substituted for another type of copper transport system (Cus), which might have something to do with the copper availability difference at neutral (pH 7) and highly alkaline (pH 10) growth conditions of halophilic and haloalkaliphilic SOB species, respectively.

### Genome Comparison of *Thiohalobacter thiocyanaticus* HRh1^T^ and “*Thiohalobacter thiocyanaticus*” FOKN1

Recently, a genome announcement has been published (Oshiki et al., 2017) and a genome deposited in the GenBank (assembly GCF_002356355) of a bacterium named as “*Thiohalobacter thiocyanaticus*” strain FOKN1 which has the 16S rRNA gene identity of 99.1% to the type strain HRh1^T^. While such values in general are typical of closely related prokaryotes, the publishing of an organism with the same species name can not be considered as legal before a full polyphasic taxonomic description is provided. Furthermore, a whole genome comparison using Mauve showed that despite the high 16S rRNA gene identity and the highly similar structure of operons and RNAs, the nucleotide pairwise identity of common regions was only 57% after alignment. This and the calculated ANI and DDH values between the two strains clearly demonstrate (Supplementary Figure S7) that strain FOKN1 represents a different genetic species and is wrongly announced as “*Thiohalobacter thiocyanaticus*.”

Therefore, here we provide only a brief comparison of the sulfur oxidation related genes present in the genome of this bacterium, since none of the genomic information is yet backed up by the published phenotypical data. Strain FOKN1 has a very similar genomic content related to its sulfur oxidation pathways with the type strain of *Thiohalobacter thiocyanaticus* HRh1^T^, including the following: FccAB type of sulfide dehydrogenase (FOKN1_0725/0726); an incomplete Sox pathway including SoxBAZYX (FOKN1_1148-1152), followed by sulfur transferases rhodanese/TusA (FOKN1_1154-1155); reversed DSR pathway for sulfur oxidation with a duplication of the key functional genes *dsrAB* (the only significant difference from the type strain), including DsrA1B1 (FOKN1_1306/1307), DsrC1C2A2B2 (FOKN1_1941/1943/1945/1946), sulfur relay proteins TusECB-DsrC (FOKN1_1947-1950), and Dsr accesory proteins DsrMKJPO (FOKN1_ FOKN1_1951-1955). For sulfite oxidation it has AprMBA (FOKN1_1661-1663)/Sat (FOKN1_1877) and a similar to HRh1^T^ putative SoeCBA (FOKN1_0490-0492).

## Conclusion

Comparative genome analysis of halophilic SOB from two distant clusters of *Gammaproteobacteria* capable of thiocyanate oxidation by the cyanate pathway demonstrated a remarkable difference in their mainstream sulfur oxidation pathway. While the *Thiohalobacter*, a member of *Chromatiales*, is oxidizing zero-valent sulfur using the rDSR pathway, common for anaerobic SOB, and thiosulfate by an incomplete Sox pathway, common to most of the aerobic gammaproteobacterial SOB, the *Guyparkeria* SCN-R1 is using complete Sox pathway, normally present in aerobic alpha- and betaproteobacteria. On the other hand, the system responsible for thiocyanate oxidation to cyanate and zero-valent sulfur is very similar in both organisms with the central role of TcDH homologous to the one present in the genus *Thioalkalivibrio*. However, the copper acquisition part of the TcDH operon of the neutrophilic halophilic SOB is clearly different from the one present in haloalkaliphilic SOB. It might be speculated, that this can be related to the different pH environment.

## Author Contributions

ST and TT performed bioinformatic analysis of the thiocyanate-related metabolism. DS performed the microbiological experiments and bioinformatic analysis of central metabolism. GM performed the phylogenetic and genome analyses and general coordination of the manuscript writing. VP participated in data analysis and writing the manuscript.

## Conflict of Interest Statement

The authors declare that the research was conducted in the absence of any commercial or financial relationships that could be construed as a potential conflict of interest.

## Footnotes

1 http://rast.nmpdr.org/

2 http://www.cbs.dtu.dk/services/TMHMM/

3 https://blast.ncbi.nlm.nih.gov/Blast.cgi

4 http://jspecies.ribohost.com/jspeciesws/

5 http://ggdc.dsmz.de/ggdc.php
