# Supplementary material for: Comparative Genomics of Thiohalobacter thiocyanaticus HRh1T and Guyparkeria sp. SCN-R1, Halophilic Chemolithoautotrophic Sulfur-Oxidizing Gammaproteobacteria Capable of Using Thiocyanate as Energy Source
Source: Front Microbiol. 2019 May 1;10:898. doi: 10.3389/fmicb.2019.00898 (PMC6504805; doi:10.3389/fmicb.2019.00898)
Supplement: Supplementary file 1 [file Data_Sheet_1.PDF]

Supplementary Material

Comparative genomics of *Thiohalobacter thiocyanaticus* and *Guyparkeria* sp. SCN-R1, halophilic chemolithoautotrophic sulfur-oxidizing *Gammaproteobacteria* capable of using thiocyanate as energy source

Stanislav V. Tsallagov, Dimitry Y. Sorokin, Tamara V. Tikhonova, Vladimir O. Popov, Gerard Muyzer[4*]

*** Correspondence:** *g.muijzer@uva.nl*

Table S1. Functional annotation of the genome of *Guyparkeria* sp. SCN-R1.

Table S2. Functional annotation of the genome of *Thiohalobacter thiocyanaticus* HRh1^T.

Fig. S1. Maximum likelihood tree of SoeA (sulfite:quinone oxidoreductase subunit A) sequences showing position of a SoeA-like protein encoded in the genome of *Thiohalobacter thiocyanaticus* HRh1^T.

Fig. S2. Dithionite-reduced minus O_2-oxidized cytochrome spectra of cell membranes from *Thiohalobacter thiocyanaticus* grown at 1 M NaCl.

Fig. S3. A fragment of multiple alignment of sequences of the C-subunite of ATP synthases from *Guyparkeria* SCN-R1 and *Thiohalobacter thiocyanaticus* HRh1^T and their homologs.

Fig. S4. Comparative SDS-PAGE (5-15%) of soluble (**Sol**) and membrane (**Mb**) fractions from cells of *Thiohalobacter thiocyanaticus* HRh1^T grown either on thiocyanate or thiosulfate as energy. source.

Fig. S5. A fragment of multiple alignment of thioredoxin-like protein sequences from *Guyparkeria* SCN-R1 and *Thiohalobacter thiocyanaticus* and their homologs.

Fig. S6. Posterior probabilities of transmembrane helix predicted by TMHMM2.0 server in the third protein encoded downstream to TcDH.

Fig. S7. Genomic comparison between *Thiohalobacter thiocyanaticus* HRh1^T and *Thiohalobacter* sp. strain FOKN1.

Table S1. Functional annotation of the genome of *Guyparkeria* sp. SCN-R1.

Gene symbol	Description	Locus tag	Celluar localization	Homolog with known function	Homolog accession number	% identity
**FCC**						
cytC	cytochrome c class I (monoheme c)	D5687_07270	TM	Cytochrome subunit of sulfide dehydrogenase	P20958	44
fcc	Sulfide dehydrogenase [flavocytochrome C] flavoprotein chain	D5687_07275	CP	Sulfide dehydrogenase [flavocytochrome *c*] flavoprotein chain	Q06530	51
**Sox system**						
soxB	Sulfur oxidation protein SoxB	D5687_09750	CP	Sulfate thiohydrolase SoxB	2WDC_A	33
soxD	Sulfite dehydrogenase diheme cytochrome c subunit SoxD	D5687_00820	CP	Sulfane dehydrogenase SoxCD	2XTS_B	34
soxC	Sulfur oxidation molybdopterin SoxC	D5687_00825	CP	Chain A, Sulfane dehydrogenase SoxCD	2XTS_A	46
soxY	Sulfur oxidation protein SoxY	D5687_01755	TM	Sulfur carrier protein Soxy	2NNC_A	52
soxZ	Sulfur oxidation protein SoxZ	D5687_01760	CP	thiosulfate oxidation carrier complex protein SoxZ	C1DB52	40
soxA	sulfur oxidation protein SoxA	D5687_05410	PP	SoxAX cytochrome complex subunit A	Q8KDM7	39
soxX	Sulfur oxidation protein SoxX	D5687_05415	TM	SoxAX	1H31_B	28
soxW	thioredoxin SoxW	D5687_05545	TM	Thiol:disulfide interchange protein DsbD;	Q6D9J6	28
**Sulfur transferases/rhodaneses**						
Tst	Sulfur transferases/rhodaneses	D5687_07715	CP	Putative thiosulfate sulfurtransferase	P27477	26
Tst	Sulfur transferases/rhodaneses	D5687_08790	TM	rhodanese	OYY31733.1	75
Tst	Sulfur transferases/rhodaneses	D5687_10700	PP	Putative thiosulfate sulfurtransferase	P16385	31
**Respiratory chain**						
ccoN2	Cytochrome c oxidase subunit CcoN 2	D5687_09640	TM	cbb_3-type cytochrome c oxidase subunit CcoN1;	D9IA43	60
ccoO1	Cytochrome c oxidase subunit CcoO 1	D5687_09645	TMM	cbb_3 cytochrome c oxidase CcoO1	3MK7_B	51
ccoP	Cytochrome c oxidase subunit CcoP	D5687_09655	TM	cbb_3-type cytochrome *c* oxidase subunit CcoP	D3NRE7	39
ccoG	Type cbb3 cytochrome oxidase biogenesis protein CcoG	D5687_09660	TM	cbb_3-type cytochrome *c* oxidase subunit CcoG	P18396.1	45
ccoI	Type cbb3 cytochrome oxidase biogenesis protein CcoI; Copper-translocating P-type ATPase	D5687_09670	TM	cbb_3-type cytochrome *c* oxidase subunit CcoI	P32113	33

ccoO2	CcoO 2	D5687_04270	TM	cbb_3-type cytochrome *c* oxidase subunit CcoO2	3MK7_B	41
ccoN2	CcoN 3	D5687_04275	TM	cbb_3-type cytochrome c oxidase subunit CcoN2	D9IA43	32
ccoS	Type cbb3 cytochrome oxidase biogenesis protein CcoS, involved in heme b insertion	D5687_03455	TM	cbb_3-type cytochrome oxidase assembly protein CcoS	WP_025367652.1	57
ccoN3	CcoN 1	D5687_09275	TM	cbb_3-type cytochrome *c* oxidase subunit CcoN3	D9IA43	58
coxB/cyoA	Cytochrome c oxidase polypeptide II (CoxB/CyoA)	D5687_06885	TM	Cytochrome *c* oxidase subunit 2	P08306	37
coxA/cyoB	Cytochrome c oxidase polypeptide I (CoxA/CyoB)	D5687_06890	TM	cytochrome *c* oxidase subunit 1	Q1RI42	53
**Carbon fixation**						
csoS1 A	CsoS1 A	D5687_04005	CM	Major carboxysome shell protein 1B	P45690	83
csoS1 B	CsoS1 B	D5687_04010	CM	Major carboxysome shell protein 1C	P45688	98
csoS1 C	CsoS1 C	D5687_04015	CM	Major carboxysome shell protein 1A	P45689	98
ccmM	carboxysome peptide B	D5687_04020	CM	carboxysome peptide B	WP_019571977.1	75
ccmL	carboxysome peptide A	D5687_04025	CM	Carboxysome shell Protein	2RCF_A	83
csoS3	CsoS3	D5687_04030	CM	carboxysome shell polypeptide	AAC32551.1	51
csoS2	CsoS2	D5687_04035	CM	carboxysomal carbonic anhydrase	2FGY_A	61
rbcM	cbbM	D5687_04040	CM	Ribulose bisphosphate carboxylase small chain	P45686	74
rbcL	cbbL formI (green)	D5687_04045	CM	Ribulose bisphosphate carboxylase large chain;	O85040	95
rbcR	CbbR RuBisCO operon transcriptional regulator	D5687_04050	CM	RuBisCO operon transcriptional regulator	P25544	55
**other Calvin cycle enzymes**						
Tkt	Transketolase	D5687_10185	CP	Transketolase 1; Short=TK 1	Q9KUP2	70
GAPDH	NAD-dependent glyceraldehyde-3-phosphate dehydrogenase	D5687_10190	CP	Glyceraldehyde-3-phosphate dehydrogenase; Short=GAPDH	P52694	77
PGK	Phosphoglycerate kinase	D5687_10195	CP	Phosphoglycerate kinase	Q1GZ23	77
Fba2	Fructose-bisphosphate aldolase class II	D5687_10205	CP	Fructose-bisphosphate aldolase; Short=FBPA;	O87796	73
PRK	Phosphoribulokinase	D5687_01665	CP	Phosphoribulokinase; Short=PRK	P23015	62
rpe	Ribulose-phosphate 3-epimerase	D5687_01950	CP	Ribulose-phosphate 3-epimerase	P44756	72
TPI	Triosephosphate isomerase	D5687_05205	CP	Triosephosphate isomerase; Short=TPI	Q3J827	57
rpiA	Ribose 5-phosphate isomerase A	D5687_05790	TP	Ribose-5-phosphate isomerase A	Q0ACJ4	69
fba1	Fructose-bisphosphate aldolase class I (bifunctional can work as	D5687_06030	CP	Fructose-bisphosphate aldolase class 1	Q8P5Z7	62

	sedoheptulose-bisphosphate aldolase)					
fbp1	Fructose-1,6-bisphosphatase, type I (bifunctional can work as sedoheptulose-bisphosphate phosphatase)	D5687_02015	CP	Fructose-1,6-bisphosphatase class 1;	A1WZH0	63
**Cyanate metabolism**						
cynA	Cyanate ABC transporter, ATP-binding protein	D5687_04980	CP	Bicarbonate transport ATP-binding protein CmpD	Q55463	49
cynB	Cyanate ABC transporter, permease protein	D5687_04985	TM	Nitrate transport permease protein NrtB	P73451	44
cynC	Cyanate ABC transporter, substrate binding	D5687_04990	CP	Nitrate transport ATP-binding protein NrtC	P73450	33
cyn	Cyanate hydratase (cyanase)	D5687_05025	CP	Cyanate hydratase; Short=Cyanase	A6W1Q1	61
ACA	alpha-Carbonic anhydrase	D5687_05030	PP	Carbonic anhydrase alpha	O52538	45
GCA	gamma-carbonic anhydrase	D5687_08965	CP	gamma-class carbonic anhydrase family	P0A9W9	47
**F1F0 ATP synthase**						
atpC	ATP synthase epsilon chain	D5687_02835	CP	ATP synthase epsilon chain	B8GRB7	42
atpD	ATP synthase beta chain	D5687_02840	CP	ATP synthase subunit beta	B8GRB8	86
atpG	ATP synthase gamma chain	D5687_02845	CP	ATP synthase gamma chain	A6W3S9	57
atpA	ATP synthase alpha chain	D5687_02850	CP	ATP synthase subunit alpha	Q1LHK8	75
atpH	ATP synthase delta chain	D5687_02855	CP	ATP synthase subunit delta	B8GRC1	49
atpF	ATP synthase F0 sector subunit b	D5687_02860	TM	ATP synthase subunit b:	Q31DL6	53
atpE	ATP synthase F0 sector subunit c	D5687_02865	TM	ATP synthase subunit c	Q31DL5	87
atpB	ATP synthase F0 sector subunit a	D5687_02870	TM	ATP synthase subunit a	A8G1X1	54
**Na/H antiporter**						
shaG	Na(+) H(+) antiporter subunit G	D5687_07980	TM	Probable K(+)/H(+) antiporter subunit G	Q9Z3Q3	38
shaF	Na(+) H(+) antiporter subunit F	D5687_07985	TM	Probable K(+)/H(+) antiporter subunit F	Q52983	44
shaE	Na(+) H(+) antiporter subunit E	D5687_07990	TM	Na(+)/H(+) antiporter subunit E1	P60688	28
shaD	Na(+) H(+) antiporter subunit D	D5687_07995	TM	Na(+)/H(+) antiporter subunit D	Q9RGZ2	35
shaC	Na(+) H(+) antiporter subunit C	D5687_08000	TM	Probable K(+)/H(+) antiporter subunit C	Q52980	53
shaAB	Na(+) H(+) antiporter subunits AB	D5687_08005	TM	Probable K(+)/H(+) antiporter subunit A/B;	Q52978	46
**Osmotic adaptation**						
opuD	Glycine betaine transporter OpuD	D5687_09280	TM	Glycine betaine transporter OpuD	P54417	46

**ectoine synthesis operon ectABC**						
ectC	L-ectoine synthase ectC	D5687_03645	CP	L-ectoine synthase	Q9AP33	62
ectB	Diaminobutyrate-pyruvate aminotransferase ectB	D5687_03650	CP	Diaminobutyrate--2-oxoglutarate transaminase	Q7M9K2	54
ectA	L-2,4-diaminobutyric acid acetyltransferase ectA	D5687_03655	CP	L-2,4-diaminobutyric acid acetyltransferaseShort=DABA acetyltransferase	O06059	44
**Sucrose-trehalose synthesis**						
TPS1	alpha-trehalose-phosphate synthase 1	D5687_07445	CP	Sucrose-6 phosphatase1	1S2O_A	29
TPS2	Alpha,alpha-trehalose-phosphate synthase 2	D5687_02105	CP	Sucrose-6 phosphatase 2	Q3J7M5	46
SPS1F	Sucrose phosphate synthase	D5687_02110	CP	Sucrose-phosphate synthase	P31927	49
INV	neutral invertase	D5687_02115	CP	Alkaline/neutral invertase A, mitochondrial;	Q9FXA8	47
**TCDH**						
yhaC	Pentapetide protein	D5687_03355	PP	Fusion of Np275 And Np276, Pentapeptide Repeat Proteins from *Nostoc punctiforme*	2J8K_A	39
SOD	Superoxide Dismutase	D5687_03345	PP	Superoxide dismutase C	2WWN_A	49
tcdh	Thiocyanate dehydrogenase	D5687_03340	PP	Thiocyanate dehydrogenase from *Thioalkalivibrio paradoxus* ARh1	5F30_A	37
-	Thioredoxin like protein	D5687_03335	PP	TlpA family protein disulfide reductase	WP_125181026.1	48
-	Sigma 54 transcriptional activator	D5687_03330	CP	Sigm54 Activator	1NY5_A	41
-	Membrane bound histidine kinase	D5687_03325	TM	histidine kinase	OYY62640	40
cusA	CusA	D5687_03320	TM	CusA	3K07_A	32
cusB	CusB-1	D5687_03315	PP	secretion protein HlyD	OJZ16289.1	40
cusB	CusB-2	D5687_03310	PP	HlyD family efflux transporter periplasmic adaptor subunit [*Thiobacillus thioparus*]	WP_018509490.1	43

[1]CP, cytoplasmic; PP, periplasmic; TM, transmembrane

Table S2. Functional annotation of the genome of *Thiohalobacter thiocyanaticus* HRh1^T.

Gene symbol	Description	Locus tag	Celluar localization[1]	Homolog with known function	Homolog accession number	% identity
			**FccAB**			
fccA	Sulfide dehydrogenase [flavocytochrome C] flavoprotein	D6C00_05870	CP	Sulfide dehydrogenase [flavocytochrome *c*] flavoprotein chain	Q06530	52
fccB	cytochrome c class I (monoheme)	D6C00_05875	TM	Cytochrome subunit of sulfide dehydrogenase	Q8KAS5	63
			**Sox system**			
soxH	SoxH protein	D6C00_12485	PP	SoxH protein	GAW75230	43
soxW	thioredoxin SoxW	D6C00_14040	PP	Thioredoxin-fold protein	4FYB_B	26
	Rhodanese domain protein	D6C00_03730	CP	Sulfur-mobilizing rhodanese-like protein	D3RPB9	50
soxX	SoxX	D6C00_03745	CP	SoxX	1H31_B	33
soxY	SoxY	D6C00_03750	TM	SoxY	2NNC_A	44
soxZ	SoxZ	D6C00_03755	CP	SoxZ	2OXH_Z	41
soxA	SoxA	D6C00_03760	PP	SoxA	Q8RLX0	56
soxB	SoxB	D6C00_03765	CP	Sulfate thiohydrolase SoxB	2WDC_A	39
soxH	SoxH protein	D6C00_15065	PP	SoxH-like protein	EGV49958.1	59
			**rDSR system**			
dsrC	DsrC	D6C00_14360	CP	sulfur relay protein DsrC	WP_114278802.1	64
dsrA	dsrA	D6C00_14370	CP	Sulfite reductase, dissimilatory-type subunit alpha	O33998	81
dsrB	dsrB	D6C00_14375	CP	Sulfite reductase, dissimilatory-type subunit beta	Q59110	44
dsrM	DsrMKJOP_ DsrM (= HmeC)	D6C00_14400	TM	Hdr-like menaquinol oxidoreductase cytochrome b-like subunit	O29749	35
dsrK	DsrMKJOP_DsrK (=HmeD)	D6C00_14405	CP	Hdr-like menaquinol oxidoreductase iron-sulfur subunit 2	O29748	43
dltD	Protein similar to glutamate synthase [NADPH] small chain, clustered with sulfite reductase	D6C00_14410	CP	Glutamate synthase [NADPH] small chain	P9WN18	31
dsrJ	DsrMKJOP_multiheme protein?? DsrJ (=HmeF) there is only a single heme there	D6C00_14415	CP	Hdr-like menaquinol oxidoreductase cytochrome c subunit	O29747	30
dsrO	DsrMKJOP iron-sulfur protein DsrO (=HmeA)	D6C00_14420	TM	Hdr-like menaquinol oxidoreductase iron-sulfur subunit 1	O29751	39
dsrP	DsrMKJOP_DsrP (= HmeB)	D6C00_14425	TM	Hdr-like menaquinol oxidoreductase	O29750	26

dsrC2/tusE	Dsr gamma - DsrC2/TusE	D6C00_03120	CP	Sulfurtransferase TusE	Q32HT7	42
dsrC3/tusE	DsrC3/TusE	D6C00_05110	CP	Sulfite reductase, dissimilatory-type subunit gamma	P45573	36
dsrC1tusE	putative: Dsr gamma subunit DsrC1/could be sulfur transferase/TusE	D6C00_14490	CP	Sulfurtransferase TusE	Q5PGB9	45
**AprABM**						
*aprA**	Adenylyl-sulfate reductase subunit A	D6C00_01360	CP	Adenylyl-sulfate reductase subunit A	O33997	94
aprB	Adenylyl-sulfate reductase subunit B	D6C00_01365	CP	Adenylyl-sulfate reductase subunit B	T2G899	44
aprM	Adenylyl-sulfate reductase membrane anchor	D6C00_01365	TM	Adenylyl-sulfate reductase membrane anchor *(Allochromatium vinosum*)	Q9RB52	62
Sat						
sat	Sulfate adenylyl transferase	D6C00_00180	CP	Sulfate adenylyl transferase	Q5PGB9	45
**SoeABC-like complex**						
*soeA***	Sulfite dehydsrogenase SoeA	D6C00_06975	PP	Putative sulfite dehydrogenase subunit A	WP_120795598	75
soeB	Sulfite dehydsrogenase SoeB ferridoxine	D6C00_06980	PP	Putative sulfite dehydrogenase subunit B	D3RNN7	79
soeC	Sulfite dehydsrogenase SoeC	D6C00_06985	TM	Putative sulfite dehydrogenase subunit C	D3RNN6	57
tst	Thiosulfate sulfurtransferase, rhodanese	D6C00_13690	CP	Putative thiosulfate sulfurtransferase	D4GYM0	31
**Hydrogenase complex**						
hdrD_5	FeS-binding domain -Hdr	D6C00_13600	CP	CoB--CoM heterodisulfide reductase iron-sulfur subunit D	P96797	28
hupB	Uptake hydrogenase large subunit1	D6C00_13605	CP	Uptake hydrogenase large subunit	P18636	59
hydB	Ni,Fe hydrogenase, subunit beta	D6C00_13615	CP	Sulfhydrogenase 1 subunit beta	Q8U2E5	33
TauD	TauD-taurine dioxygenase	D6C00_14705	CP	Taurine catabolism dioxygenase TauD	WP_012638742	57
hydG	cytochrome-c3 hydrogenase gamma chain	D6C00_13620	CP	Sulfhydrogenase 1 subunit gamma	Q8U2E4	34
hydD		D6C00_13625	CP	Sulfhydrogenase 1 subunit delta	WP_126605181	67
**Cyanate metabolism**						
Cyn	Cyanate hydratase	D6C00_04640	CP	Cyanate hydratase	A6W1Q1	63
cynA	Cyanate ABC transporter, ATP-binding protein	D6C00_04645	CP	Bicarbonate transport ATP-binding protein CmpD	Q55463	47
GCA	Gamma-carbonic anhydrase	D6C00_08200	CP	Gamma carbonic anhydrase 1	Q9FWR5	43

BCA	Beta-carbonic anhydrase	D6C00_09370	CP	Carbonic anhydrase 2	P9WPJ8	38
CAH	OI-carbonic anhydrase (3)	D6C00_12350	CP	Carbonic anhydrase	5ZTP_A	47
CAH	OI-carbonic anhydrase (1)	D6C00_04625	CP	Carbon disulfide hydrolase	S5FT07	77
**RuBisCo**						
CsoS1 A	carboxysome shell protein CsoS1 (A)	D6C00_05915	CP	Carbon dioxide-concentrating mechanism protein CcmK	P0A328	97
CsoS1 B	carboxysome shell protein CsoS1 (B)	D6C00_05920	CP	Carbon dioxide-concentrating mechanism protein CcmK	Q7V2D1	95
CsoS1 C	carboxysome shell protein CsoS1 (C)	D6C00_05925	CP	Carbon dioxide-concentrating mechanism protein CcmK	P0A328	92
CcmM	carboxysome peptide B	D6C00_05930	CP	Carboxysome Shell Protein	2RCF_A	48
ccmL	carboxysome peptide A	D6C00_05935	CP	Carboxysome Shell Protein	2RCF_A	70
CsoS3	carboxysome shell protein CsoS3	D6C00_05940	CP	Beta Carbonic Anhydrase	2FGY_A	45
CsoS2	carboxysome shell protein CsoS2	D6C00_05945	CP	carboxysome shell protein CsoS2	EAR23171.1	70
rbcR	RuBisCO operon transcriptional regulator CbbR	D6C00_06940	CP	RuBisCO operon transcriptional regulator	P25544	57
rbcO	Rubisco activation protein CbbO	D6C00_06955	CP	Protein NorD	Q576X0	34
rbcQ	Rubisco activation protein CbbQ	D6C00_06960	CP	Protein CbbQ	Q51858	77
rbcM	cbbM	D6C00_06965	CP	Ribulose bisphosphate carboxylase small chain	P24682	83
rbcL	cbbL	D6C00_06970	CP	Ribulose bisphosphate carboxylase large chain	Q56259	91
**Other Calvin cycle enzyme**						
PRK	phosphoribulokinase	D6C00_06745	CP	Phosphoribulokinase	P19923	66
TPI	Triosephosphate isomerase	D6C00_01165	CP	Triosephosphate isomerase	Q3J827	58
Fba1	Fructose-bisphosphate aldolase class I	D6C00_01985	CP	Fructose-bisphosphate aldolase class 1	P0A992	70
Fbp	Fructose-1,6-bisphosphatase, type V, archaeal	D6C00_02820	CP	Fructose-1,6-bisphosphate aldolase/phosphatase	Q72K02	61
Fbp1	Fructose-1,6-bisphosphatase, type I bisphosphate	D6C00_05130	CP	Fructose-1,6-bisphosphatase class 1	Q3SFS6	72
araD	Ribulose-5-phosphate 4-epimerase	D6C00_05625	CP	L-fuculose phosphate aldolase	Q58813	27
Tkt	Transketolase	D6C00_09785	CP	Transketolase 1	Q9KUP2	71
GAPDH	NAD-dependent glyceraldehyde-3-phosphate dehydrogenase	D6C00_09790	CP	Glyceraldehyde-3-phosphate dehydrogenase	P52694	80
PGK	Phosphoglycerate kinase	D6C00_09795	CP	Phosphoglycerate kinase	B8GP44	77
Fba2	Fructose-bisphosphate aldolase class II	D6C00_09805	CP	Fructose-bisphosphate aldolase	O87796	78

rpiA	Ribose 5-phosphate isomerase A	D6C00_10225	TM	Ribose-5-phosphate isomerase A	Q0ACJ4	73
**ATP synthesis**						
atpD	synthase beta chain1	D6C00_02825	CP	ATP synthase subunit beta	A8ZNR6	64
atpC	synthase epsilon chain	D6C00_02830	CP	ATP synthase epsilon chain 2	Q13IW4	37
AtpC	F-type H+-ATPase subunit AtpC	D6C00_02835	TM	F-type H+-transporting ATPase subunit AtpC	WP_006964569.1	70
NHA	Na(+)/H(+) antiporter	D6C00_02840	TM	Sodium Proton Antiporter	4CZ9_A	25
atp-1	ATP synthase subunit I	D6C00_02845	TM	ATP synthase subunit I	WP_113862237.1	53
atpB	synthase F0 subunit a1	D6C00_02850	TM	ATP synthase subunit a 2	A8ZNS1	64
atpE	Na+-ATP synthase F0 sector subunit c1	D6C00_02855	TM	ATP synthase subunit c 2	A3PS63	65
atpF	synthase F0 sector subunit b2	D6C00_02860	TM	ATP synthase subunit b 2	Q21ZA0	39
atpA	synthase alpha chain2	D6C00_02865	CP	ATP synthase subunit alpha 2	A8ZNS4	63
atpG	synthase gamma chain2	D6C00_02870	CP	ATP synthase gamma chain	B0THN3	30
VHA-I	V-type ATP synthase subunit I	D6C00_14700	TM	V-type ATP synthase subunit I	O57721	24
VHA-K	V-type ATP synthase subunit K	D6C00_14705	TM	V-type proton ATPase	Q41773	34
VHA-A	V-type ATP synthase subunit A	D6C00_14720	CP	V-type ATP synthase alpha chain	B8CZG8	49
VHA-B	V-type ATP synthase subunit B F1F0-Na-ATPase	D6C00_14725	CP	V-type ATP synthase beta chain	Q8U4A5	55
ShAB	Na(+) H(+) antiporter subunit A/B	D6C00_15020	TM	Probable K(+)/H(+) antiporter subunit A/B	Q52978	52
shaD	Na(+) H(+) antiporter subunit D	D6C00_15030	TM	Probable K(+)/H(+) antiporter subunit D	Q52981	42
shaE	Na(+) H(+) antiporter subunit E	D6C00_15035	TM	Na(+)/H(+) antiporter subunit E1	P60688	28
shaF	Na(+) H(+) antiporter subunit F	D6C00_15040	TM	Probable K(+)/H(+) antiporter subunit F	Q52983	50
shaG	Na(+) H(+) antiporter subunit G	D6C00_15045	TM	Probable K(+)/H(+) antiporter subunit G	Q9Z3Q3	45
**Osmoprotection**						
TPS	Trehalose-6-phosphate synthase	D6C00_02875	CP	Trehalose-phosphate phosphatase	Q49734	40
trePP	Trehalose 6-phosphate phosphorylase	D6C00_02880	CP	Trehalose 6-phosphate phosphorylase	Q9CID5	51
TPS	Trehalose-phosphate synthase	D6C00_10705	CP	Sucrose-phosphatase 1	Q5IH14	54
betC	Choline-sulfatase	D6C00_13525	TM	Arylsulfatase	Q8XNV1	25
ProW	L-proline glycine betaine ABC transport system permease protein	D6C00_06040	TM	Glycine betaine/carnitine transport permease protein GbuB	Q9RR45	52

	ProW					
ProV	L-proline glycine betaine ABC transport system permease protein ProV	D6C00_06045	CP	Glycine betaine/choline transport system ATP-binding protein OusV	E0SCY1	58
ProX	L-proline glycine betaine binding ABC transporter protein ProX	D6C00_06035	CP	Glycine betaine/carnitine transport binding protein GbuC	Q9RR44	36
**Sucrose biosynthesis**						
SUS	Sucrose synthase	D6C00_08230	CP	Sucrose synthase	Q820M5	60
FRK	Fructokinase	D6C00_08240	CP	Probable fructokinase-5	O82616	27
**Respiratory cytochromes**						
CcoH	CcoH	D6C00_07505	TM	cbb_3-type cytochrome c oxidase subunit CcoH	P18397, RMG59777.1	48
CcoG1	cbb_3 cytochrome oxidase biogenesis protein CcoG	D6C00_07510	TM	cbb_3-type cytochrome c oxidase subunit CcoG	P18396	41
CcoP1	Cytochrome c oxidase subunit CcoP 1	D6C00_07515	TM	cbb_3-type cytochrome c oxidase subunit CcoP	A4VKL4	51
CcoN	Cytochrome *c* cbb_3 oxidase subunit CcoN	D6C00_09260	TM	cbb_3-type cytochrome c oxidase subunit CcoN	D9IA43	61
CcoO	Cytochrome *c* oxidase subunit CcoO	D6C00_09265	TM	cbb_3-type cytochrome oxidase, chain B	3MK7_B	55
CcoP2	Cytochrome c oxidase subunit CcoP 2	D6C00_09275	TM	cbb_3-type cytochrome c oxidase subunit CooP	A8HZ17	43
CcoG2	Type cbb_3 cytochrome oxidase biogenesis protein CcoG	D6C00_09280	TM	Protein RdxB	P54932	42
CcoS	Type cbb_3 cytochrome oxidase biogenesis protein CcoS	D6C00_09405	TM	cbb3-type cytochrome oxidase assembly protein CcoS	WP_110018494.1	69
**Cytochrome c / quinol oxidase ba3**						
CoxA/CyoB	Cytochrome *c* oxidase polypeptide I (a_3 subunit)	D6C00_09220	TM	Probable cytochrome *c* oxidase subunit 1	Q4ULU5	56
CtaG	Cytochrome oxidase biogenesis protein	D6C00_09225	TM	Cytochrome *c* oxidase assembly protein CtaG	A4Z2D0	42
Cox2	Cytochrome c oxidase cytochrom *b* sunbunit polypeptide II	D6C00_09215	TM	Cytochrome *c* oxidase subunit 2	P08306	39
Cox3	Cytochrome c oxidase polypeptide III Cyt. *c* oxidase bo_3?	D6C00_09230	TM	Cytochrome *c* oxidase subunit 3	Q9ZZY5	39
CoxB/CyoA	Cytochrome c oxidase $(B(O/a)_3$-type) chain II	D6C00_10845	TM	Cytochrome *c* oxidase subunit 2	P98052	28
Cox1	Cytochrome c oxidase $(B(O/a)_3$-type) chain I	D6C00_10850	TM	Cytochrome *c* oxidase polypeptide 1	P33518	24
**TCDH**						
yhaC	Pentapetide protein	D6C00_06780	PP	Pentapeptide Repeat Protein Involved In Heterocyst Differentiation Regulation	3DU1_X	40
-	FMN-binding redoxin-containing protein	D6C00_06785	CP	redoxin domain-containing protein	WP_111384496.1	61

tcdh	Thiocyanate dehydrogenase	D6C00_06790	PP	Thiocyanate Dehydrogenase	5F30_A	34
-	Thioredoxin like protein	D6C00_06795	PP	redoxin domain-containing protein	WP_111384496.1	55
-	Sigma 54 transcriptional activator	D6C00_06800	CP	Sigm54 Activator	1NY5_A	40
-	Membrane bound histidine kinase	D6C00_06805	TM	histidine kinase [*Thiobacillus* sp. SCN 63-57]	ODU99242.1	41
CusA	CusA	D6C00_06810	TM	CusA	3K07_A	30
CusB	*CusB-1*	D6C00_06815	PP	secretion protein HlyD [Thiobacillus sp. 63-78]	OJZ16289.1	36
CusB	*CusB-2*	D6C00_06820	TM	HlyD family efflux transporter periplasmic adaptor subunit	WP_018509490.1	42

*The AprA is annotated in GenBank as frame-shifted, the gene consisted in two separate frames. The recovered protein is the following:

```
<MAYETIVEDNIDILVCGAGLGGTGAAWEARYWGQDKKIVIAEKANIDRSGAVAQGLYAINCYMGTRFGENNPEDHVRYARIDLMGMVREDLAFDMARHVDSAVHQFEEWGLPLMRDPKTGAYQREGRWQIMIHGESYKPIVAEA
AKKSADKVFNRICVTHLLMDESKENRIAGAVGFNVRTGNYHVFKSKTVIVAAGGASNIYKPRSVGEGARRVWYAPWSSGSAYGLLISAGAKMTQMENRIVLARFKDGYGPVGAYFLHLKTYTQNCLGEEYESKWWPQLQEMVGKE
YLDPEASHRTHRPIPTCLRNHALISEVNAGRGPIHMVTMEAFQDPHLEEIGWHNFLGMTVGQAVLWAATDVDPKNENPELTTSEPYVMGSHATGCGAWCSGPEDVSPPEYFWGYNRMTTVEGLFGAGDAVGGTPHAFSSGSFTEG
RLAAKAACKYIDDGKAEGIVVSQKQIEDRRKEIYKPLEHYRIYRNEITAGSVNPNYINPRQGLDRLQKLMDEYAGGASVNYMTNEKLLHIGLKKLKLLEEDFEKIAAEDIHELLRAWELKHRILSSEAVMQHTLFRKETRWPGYY
YRGDFLKVDDENWHVLTVSRRDPKTGEYTMEKAPCYHLVEDTE>
```

**Based on phylogenetic reconstruction of D6C00_06975

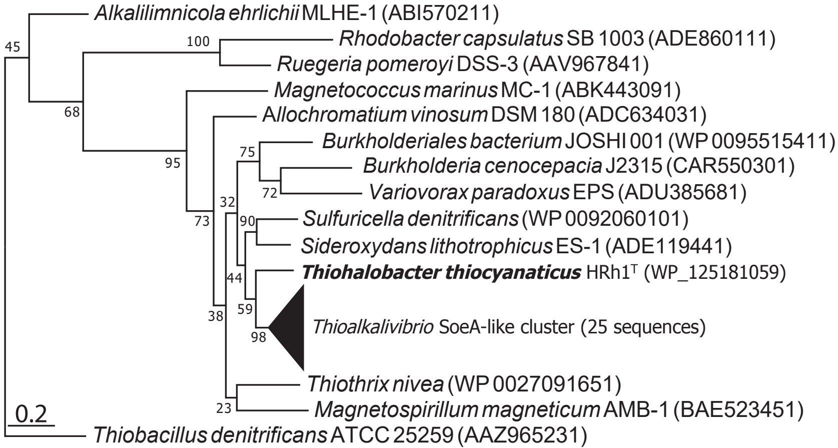

Fig.**S1**. Maximum likelihood tree of SoeA (sulfite:quinone oxidoreductase subunit A) sequences showing position of a SoeA-like protein encoded in the genome of Thiohalobacter thiocyanaticus HRh1^T. Sequences of SorA were used as outgroup and pruned from the tree. The scale bar represents sequence difference. Bootstrap values (from 500 replicates) are shown on the nods.

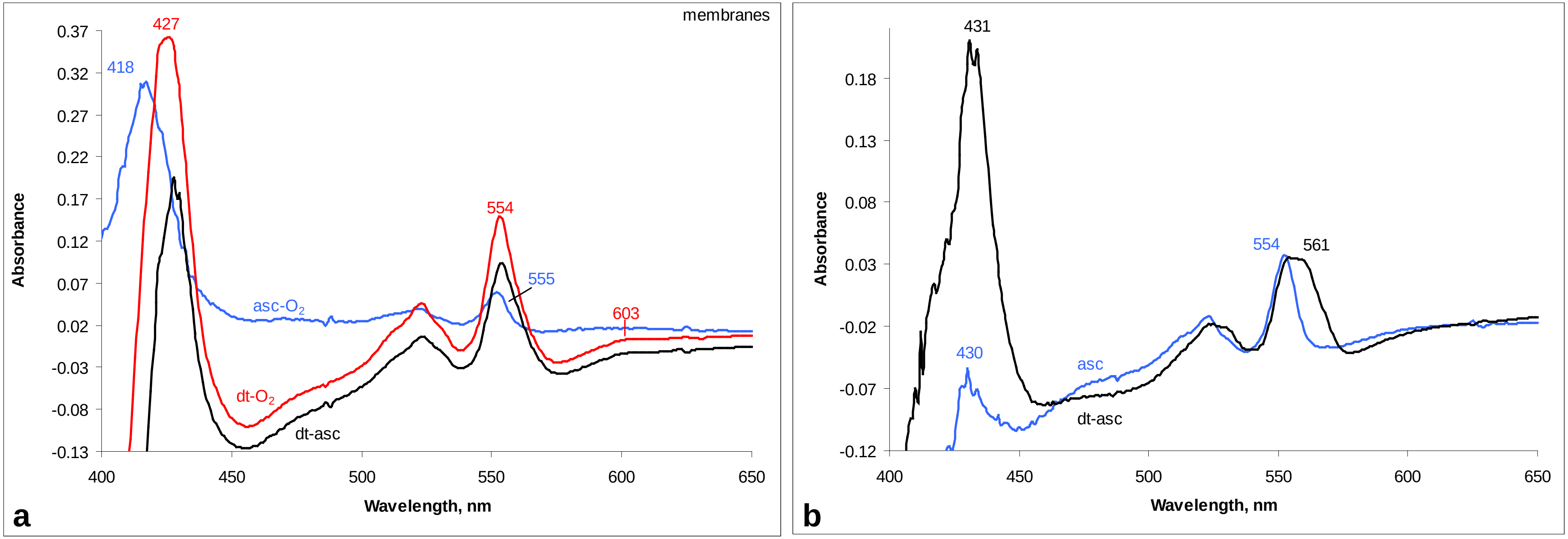

Fig. **S2**. Dithionite-reduced minus O_2-oxidized cytochrome spectra of cell membranes from Thiohalobacter thiocyanaticus grown at 1 M NaCl with either thiosulfate (**a**) or thiocyanate (**b**) as electron donor. In the thiosulfate grown cells 3 types of hemes are present: *c, b* and a_3 with a domination of heme *c*, while in the thiocyanate-grown cells only *c* and *b* hemes are detectable with a higher proportion of the heme *b* type.

(a)

(b)

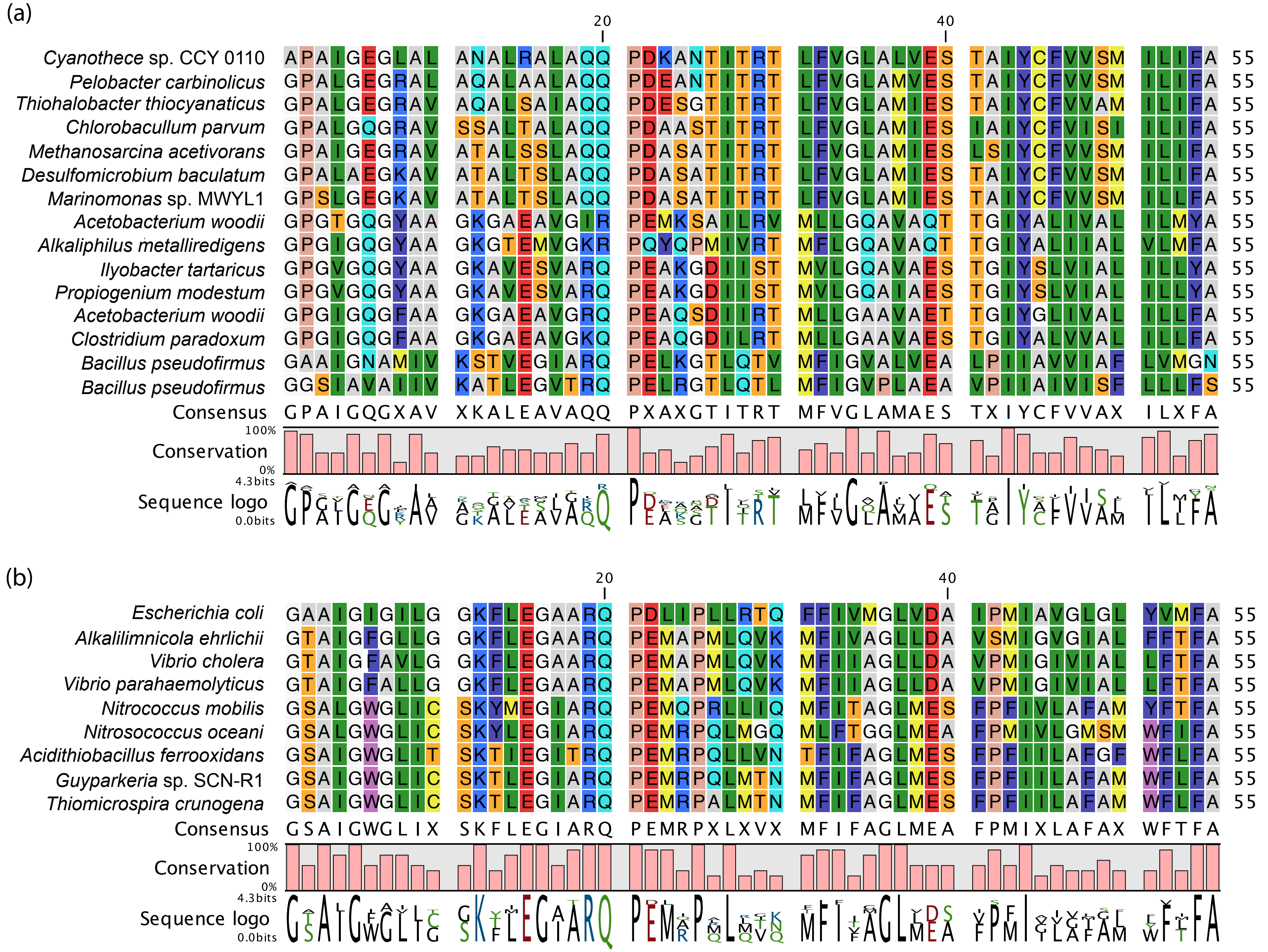

Fig. **S3**. A fragment of multiple alignment of sequences of the C-subunite of ATP synthases from *Guyparkeria* SCN-R1 and *Thiohalobacter thiocyanaticus* HRh1^{T} and their homologs (used to construct the phylogenetic tree (**Fig. 3**).

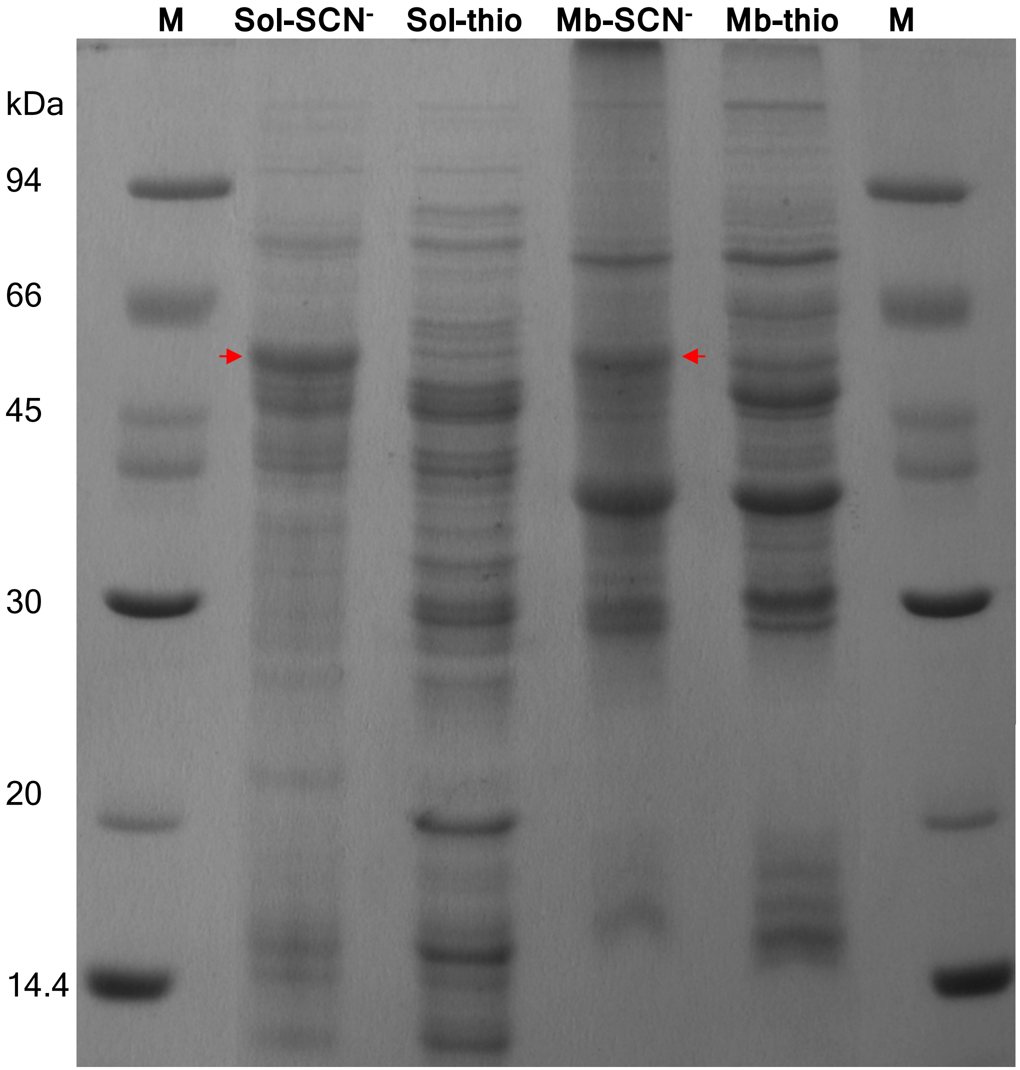

Fig. **S4**. Comparative SDS-PAGE (5-15%) of soluble (Sol) and membrane (Mb) fractions from cells of *Thiohalobacter thiocyanaticus* HRh1^T grown either on thiocyanate or thiosulfate as energy source. The arrows indicate a polypeptide differentially overexpressed during growth with thiocyanate. Protein load - 20 µg ml^{-1}.

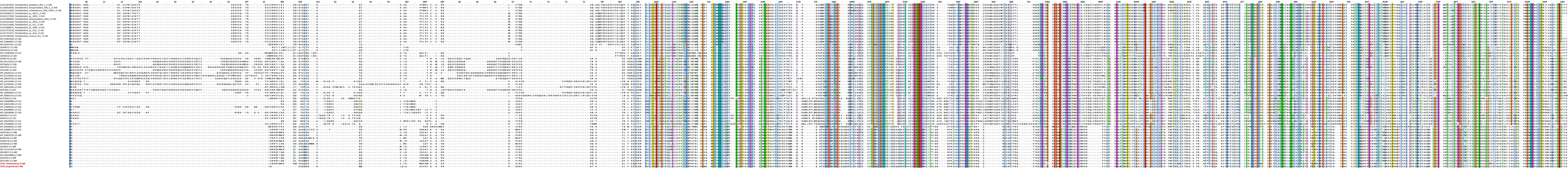

Fig. **S5**. A fragment of multiple alignment of thioredoxin-like protein sequences from *Guyparkeria* SCN-R1 and T*hiohalobacter thiocyanaticus* HRh1^T and their homologs. In four most distant homologs a CXXC motif appears and the

Fig. **S6**. Posterior probabilities of transmembrane helix predicted by TMHMM2.0 server in the third protein encoded downstream to TcDH.

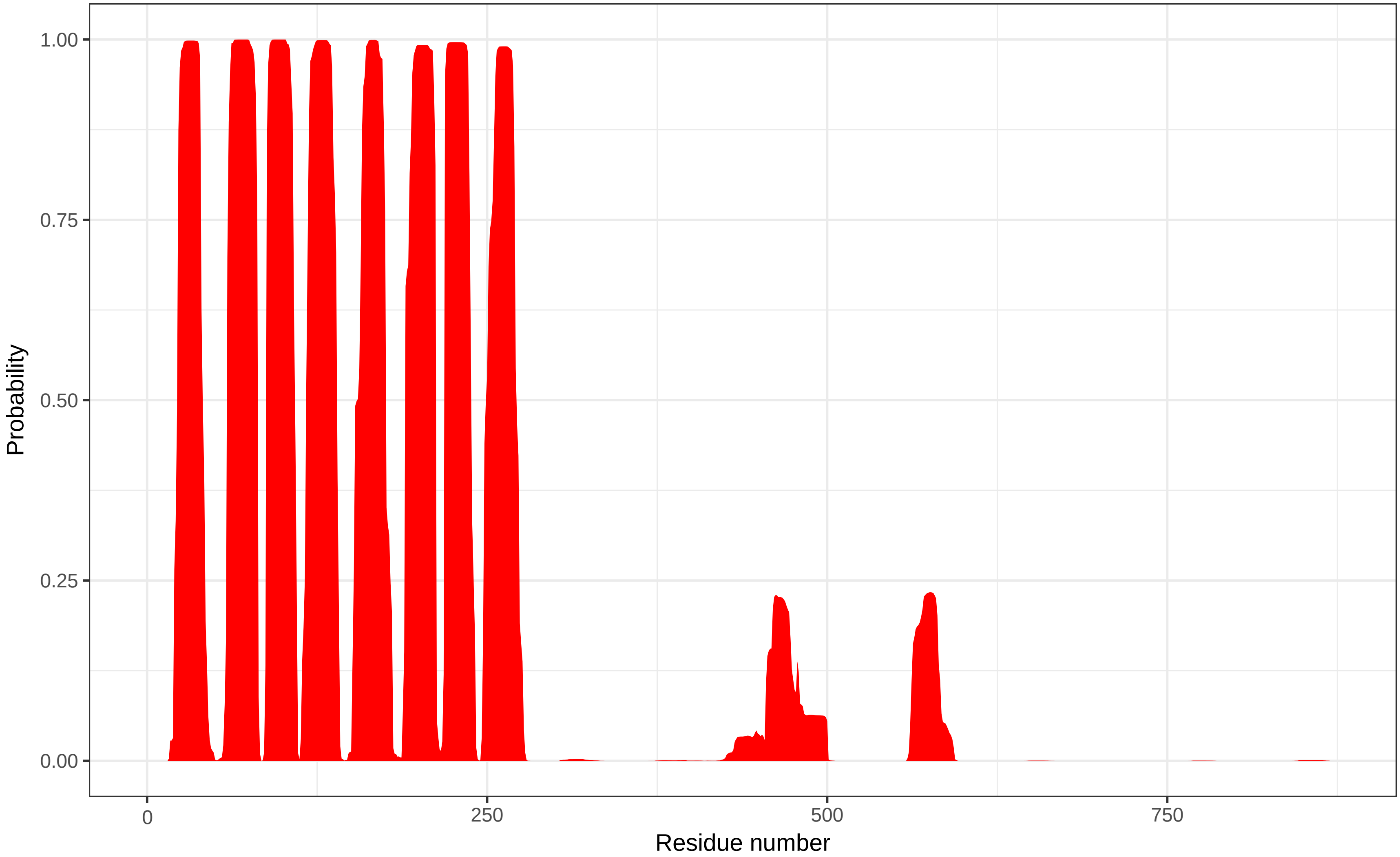

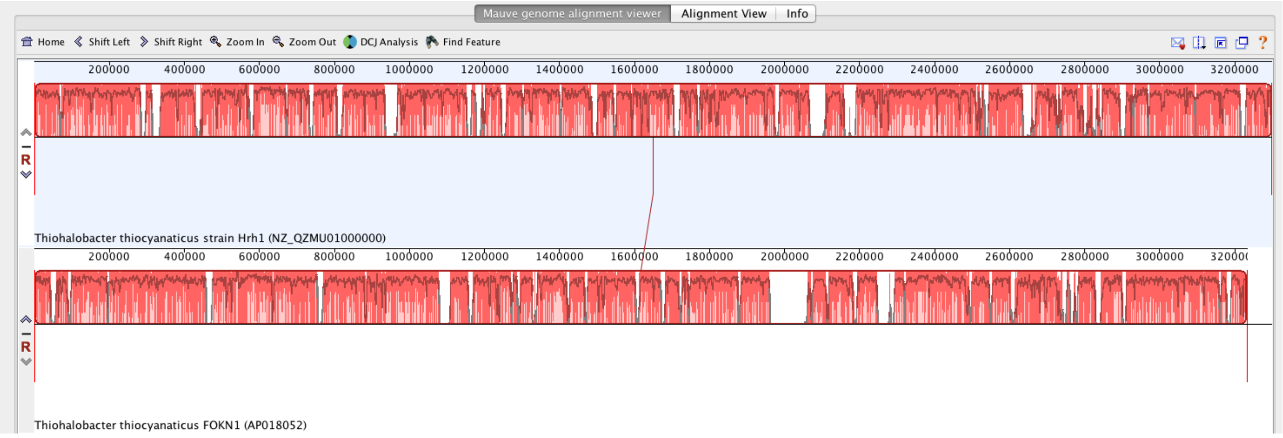

	**HRh1^{T}**	FOKN1
**ANIb** [and aligned percentage]		
Thiohalobacter thiocyanaticus HRh1	*	85.79 [70.14]
Thiohalobacter sp. strain FOKN1	86.01 [71.72]	*
**ANIm** [and aligned percentage]		
Thiohalobacter thiocyanaticus HRh1	*	88.06 [69.74]
Thiohalobacter sp. strain FOKN1	88.06 [71.18]	*
**GGDC** (**DDH formula 2**)		
Thiohalobacter thiocyanaticus HRh1	*	31.40 [29-33.9]
Thiohalobacter sp. strain FOKN1	31.40 [29-33.9]	*

Fig. **S7**. Genomic comparison between *Thiohalobacter thiocyanaticus* HRh1^{T} and *Thiohalobacter* sp. strain FOKN1.
